# Complications and side effects of Wide-Awake Local Anaesthesia No Tourniquet (WALANT) in upper limb surgery: a systematic review and meta-analysis

**DOI:** 10.1007/s00264-024-06104-9

**Published:** 2024-02-17

**Authors:** Jad Lawand, Ashraf Hantouly, Fadi Bouri, Mohammad Muneer, Abdulaziz Farooq, Elisabet Hagert

**Affiliations:** 1 Medical Branch, University of Texas, 301 University Blvd, Galveston, TX 77555 USA; 2 Department of Orthopedic Surgery, Hamad Medical Cooperation, Doha, Qatar; 3 Department of Plastic Surgery, Hamad Medical Corporation, Doha, Qatar; 4 Aspetar Orthopaedic and Sports Medicine Hospital, Doha, Qatar; 5 Department of Clinical Science and Education, Karolinska Institutet, Stockholm, Sweden

**Keywords:** Anesthesia, Hand, Trauma, Upper limb, WALANT, Wrist

## Abstract

**Purpose:**

Wide-Awake Local Anaesthesia No Tourniquet (WALANT), a groundbreaking anaesthetic technique resurging in practice, warrants a comprehensive safety analysis for informed adoption. Our study aimed to identify complications/side effects of WALANT upper limb procedures through a systematic review and meta-analysis.

**Methods:**

This PROSPERO-registered study was performed with strict adherence to PRISMA guidelines. Embase, OVIDMedline, Cochrane, Web of Science, and Scopus databases were searched until February 2023. Inclusion criteria involved English articles, reporting complications/side effects in primary WALANT upper limb surgeries. Outcomes included all complications and side effects, data on the anaesthetic mixture, publication year/location, study type, and procedures performed. The meta-analysis employed the Freeman-Tukey Double Arcsine Transformation, computed *I*^2^ statistics, and utilized common or random effects models for pooled analysis.

**Results:**

2002 studies were identified; 79 studies met the inclusion criteria representing 15,595 WALANT patients. A total of 301 patients had complications, and the meta-analysis using a random effects model provided a complication rate of 1.7% (95% CI: 0.93–2.7%). The most reported complications were superficial infection (41%, *n* = 123/300), other/specified (12%, *n* = 37/300), and recurrent disease (6.7%, *n* = 20/300). A decade-by-decade analysis revealed no statistically significant difference in complication rates spanning the last three decades (*p* = 0.42). Adding sodium bicarbonate to the anaesthetic solution significantly reduced postoperative complications (*p* = 0.025).

**Conclusion:**

WALANT has a low overall complication rate of 1.7%, with no significant temporal variation and a significant reduction in complications when sodium bicarbonate is added to the anaesthetic solution. Our findings support the safety of WALANT in upper limb procedures.

**Registration:**

PROSPERO: CRD42023404018.

**Supplementary Information:**

The online version contains supplementary material available at 10.1007/s00264-024-06104-9.

## Introduction

Healthcare systems are facing substantial challenges with increasing costs and a growing ageing population. In the USA, costs for healthcare soared over 500% in the past five decades, with the upward trend expected to persist [1]. As of 2022, the UK orthopaedic surgery waitlists surged to over 60,000 patients, up from 435 in January 2020 [2]. Wide-Awake Local Anaesthesia No Tourniquet (WALANT), as described by Lalonde, utilizes epinephrine and local anaesthetic mixtures for intra-operative injections, serving as an alternative to traditional anaesthesia [3]. This technique not only ensures effective pain control and anaesthesia but also promotes patient participation, which improves surgical outcomes [4–6].

Anesthetized patients endure a maximum tourniquet time of two h with inherent risks and limitations [7, 8]. However, WALANT eliminates tourniquet use in upper limb surgery, minimizing patient discomfort, easing resource burdens, and enhancing surgical care accessibility [9–11]. Surgeons have successfully utilized WALANT in diverse procedures, including skin flap surgery, joint replacements, upper and lower limb fracture fixations, and tendon transfers [12–15]. This technique proved crucial during the COVID-19 pandemic, preserving hospital resources and ensuring timely orthopaedic care [16–24].

However, its global adoption requires a thorough safety assessment. Presently, a lack of pooled data analysis from studies hinders the understanding of WALANT complications and side effects. This study aims to assess complications and side effects in upper limb procedures with the hypothesis that WALANT is a safe and effective technique, with a low complication rate.

## Materials and methods

This PROSPERO-registered systematic review was conducted with strict adherence to the Preferred Reporting Items for Systematic Reviews and Meta-Analyses (PRISMA) [25]. The focus was studies reporting complications and side effects with the use of WALANT technique in upper limb surgeries.

### Search strategy

Five online databases (Embase, OVIDMedline, Cochrane, Web of Science, and Scopus) were searched on 26 February 2023. All articles from inception till the search date were eligible for screening. Terms for the database search included: “walant”, “wide-awake”, “wide awake”, “wide awake no tourniquet”, “wide awake without tourniquet”, “walant”, “local anaesthetic no tourniquet”, “local anaesthetic without tourniquet” (Supplementary Fig. 1).

### Inclusion criteria

Clinical studies that reported complications and/or side effects of the WALANT technique in primary upper limb surgeries performed in adults (> 18 years).

### Exclusion criteria

Non-clinical studies, revision surgeries, case reports, conference abstracts, animal studies, cadaveric studies, technique studies, unpublished manuscripts, non–English language studies, paediatric patient population (< 18 years), and those not reporting postoperative complications/side effects.

### Study screening

Two authors (JL, FB) performed an independent and blind title and abstract screening. For the full-text review phase, two authors screened the eligible articles independently and blindly, and any discrepancy was discussed with the senior author (EH) to reach a consensus.

### Data extraction process and data items

Two authors extracted and summarized data from included articles independently, using Google Sheets (Google LLC, Mountain View, CA, USA). Data extracted were: demographics, study characteristics, follow-up period, comorbidities, local anaesthetic mixture, epinephrine dilution concentration, the use of added sodium bicarbonate, type of surgery, operative time, reoperation, conversion to general anaesthesia, and complications. The complications noted were infections, wound complications, nerve complications, and others. Infections were categorized into superficial and deep infections. Wound complications included delayed healing, wound dehiscence, and drainage. Nerve complications included digital nerve scarring and neuropraxia. Other complications included hematoma, finger necrosis, vascular injury, vasovagal response, development of complex regional pain syndrome, recurrent disease, tendon rupture, nonunion, lateral band subluxation, and unspecified.

### Statistical methodology

Our comprehensive data analysis aggregated and analysed data synthesis from individual studies using R (version 4.2.0). To gauge the prevalence of complications across the spectrum of included studies, we used meta library package from R software for conducting meta-analysis of proportions. Notably, our dataset often featured proportions that approached zero or were exactly zero. In such instances, it is considered best practice to utilize the Freeman-Tukey Double Arcsine Transformation [26]. This statistical technique not only stabilizes variance but also substantially enhances the precision of the estimated confidence intervals. We computed the *I*^2^ statistic which quantifies the proportion of variation in the study outcomes that is due to true between-study differences rather than random error. An *I*^2^ greater than 50% was regarded as the presence of substantial heterogeneity, and the random effects model rather than the common effects model was reported in the result [27]. Additionally we also did meta-analysis using subgroups and constructed informative forest plots that facilitated a comparative analysis of complication rates across several crucial variables, including age, gender, study design, publication year, geographical region, follow-up duration, lidocaine concentration (1% or 2%), epinephrine concentration, use of sodium carbonate, and the specific surgical procedures undertaken (carpal tunnel release, trigger finger release, and flexor tendon repair).

## Results

### Overall complication rate and types of complications

**Fig. 1:**
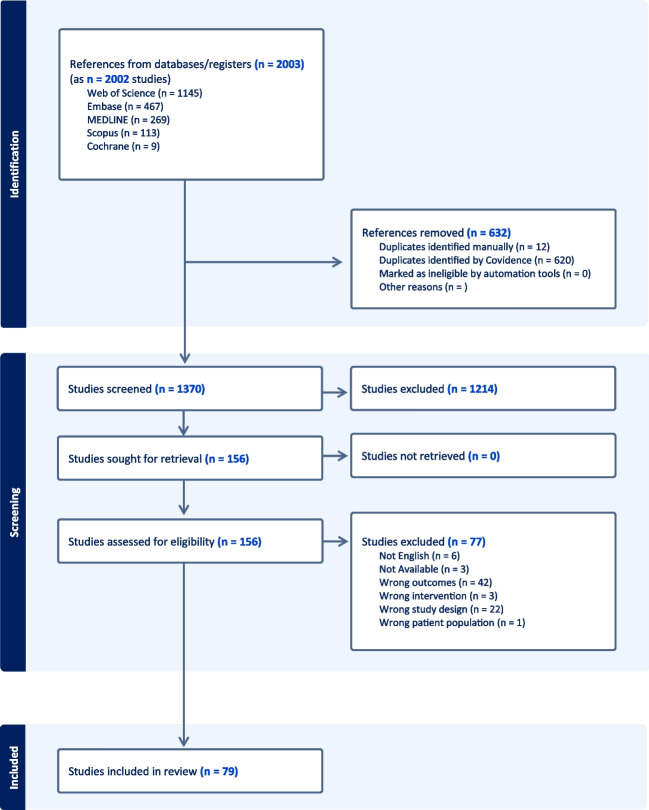
PRISMA flow chart

**Fig. 2:**
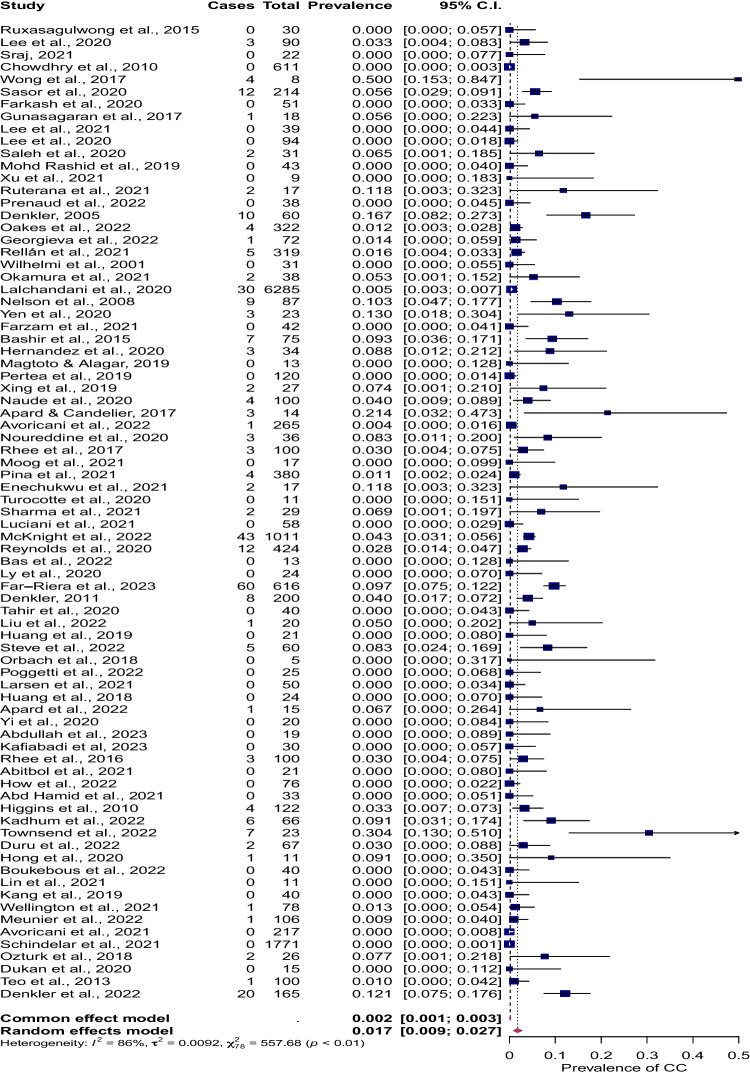
Forest plot of WALANT complication incidence

In our comprehensive analysis of 15,595 patients treated under WALANT, 79 studies were included in our analysis, as depicted in Fig. 1 PRISMA diagram [4, 9–11, 14–16, 19, 20, 28–97]. The meta-analysis using a random effect model indicates an overall complication rate of 1.7% (95% CI: 0.93–2.7%), as shown in Fig. 2. Of the complications reported, superficial infection was the most common (41%, *n* = 123/15,595) followed by other/unspecified (12%, *n* = 37/15,595), and wound dehiscence (7%, *n* = 20/15,595). Two studies reported nerve-related complications, in a total of four patients (0.02%). Three patients suffered complications following needle aponeurotomy for Dupuytren’s contracture (two neurapraxia, one permanent digital nerve injury), and the fourth was a transient painful neuroma following burn contracture release.

**Fig. 3:**
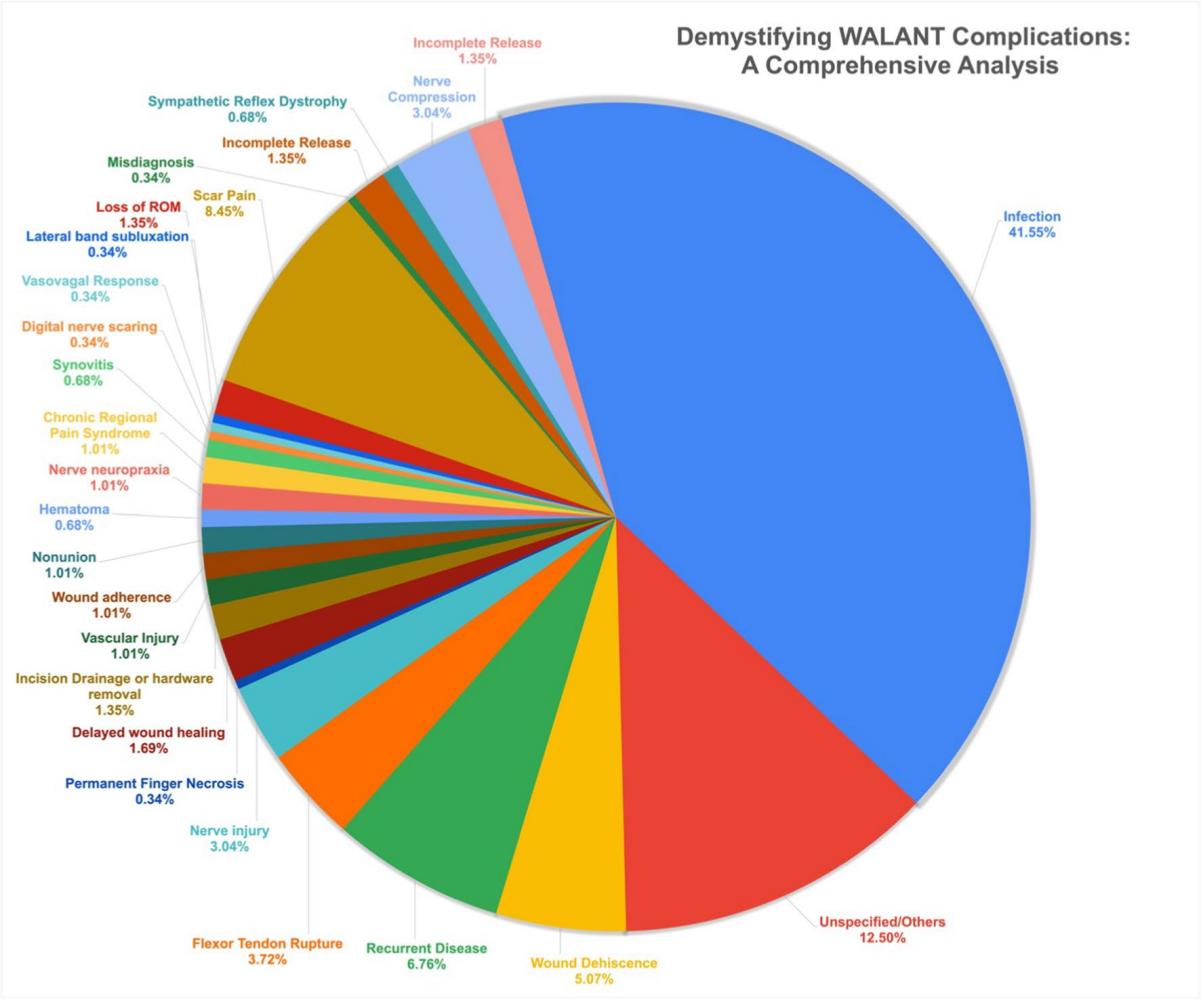
Breakdown of WALANT complications by type

We observed only one case of permanent digital ischemia requiring partial amputation amongst 15,595 patients, with a detailed complication breakdown in Fig. 3. Furthermore, there exists a notable uniformity in the prevalence of complication rates across diverse procedures ranging from carpal tunnel release (CTR; 0.05%, 95% CI: 0.0–0.23%), trigger finger release (TFR; 0.49%, 95% CI: 0.0–1.9%), and flexor tendon repair (FTR; 1.2%, 95% CI: 0.0–6.9%) as depicted in Table 1, and Supplementary Figs. 2–4.

**Table 1:** Prevalence of WALANT complications by surgical procedures

Variable	Group	*I*^2^	*K* (no. studies)	Sample size (*n*)	Number of complications	CE model (95% CI)	RE model (95% CI)
Prevalence of complications	Overall, for all procedures	86.0%	79	15,595	300	0.02% [0.0–0.23%]	1.7% [0.93–2.73%]
	Carpal tunnel release	79.4%	28	2621	72	0.2% [0.0–0.23%]	0.05% [0.0–0.85%]
	Trigger finger release	65.4%	19	1837	55	0.74% [0.25–1.4%]	0.49% [0.0–1.9%]
	Flexor tendon repair	54.8%	10	328	20	0.84% [0.0–3.2%]	1.2% [0.0–6.9%]

*CE* common effects; *RE* random effects

A subsequent subanalysis, comparing elective procedures (CTR, TFR) to traumatic procedures (FTR), identified a significant difference in complication rates, with traumatic procedures showing a higher rate of complications (*p* = 0.036), as illustrated in Supplementary Fig. 5.

### Complications over time

A decade-by-decade analysis of the studies included revealed no statistically significant difference in complication rates spanning the last three decades (*p* = 0.42; see Supplemental Fig. 6).

### Study design and complications reported

When studies were grouped into retrospective and prospective study design groups, it was found that prospective studies reported statistically higher rates of complications as compared to retrospective studies (*p* = 0.033; see Supplemental Fig. 7).

### Complication rate in relation to age

In a subgroup analysis that classified included studies by mean age of participants into three age groups: < 45, 45–65, and > 65 years, encompassing 20, 30, and 7 studies, respectively, no statistically significant differences in complication rates were noted (*p* = 0.58). The forest plot is available in Supplemental Fig. 8.

### Geographical distribution of publications and complications

**Fig. 4:**
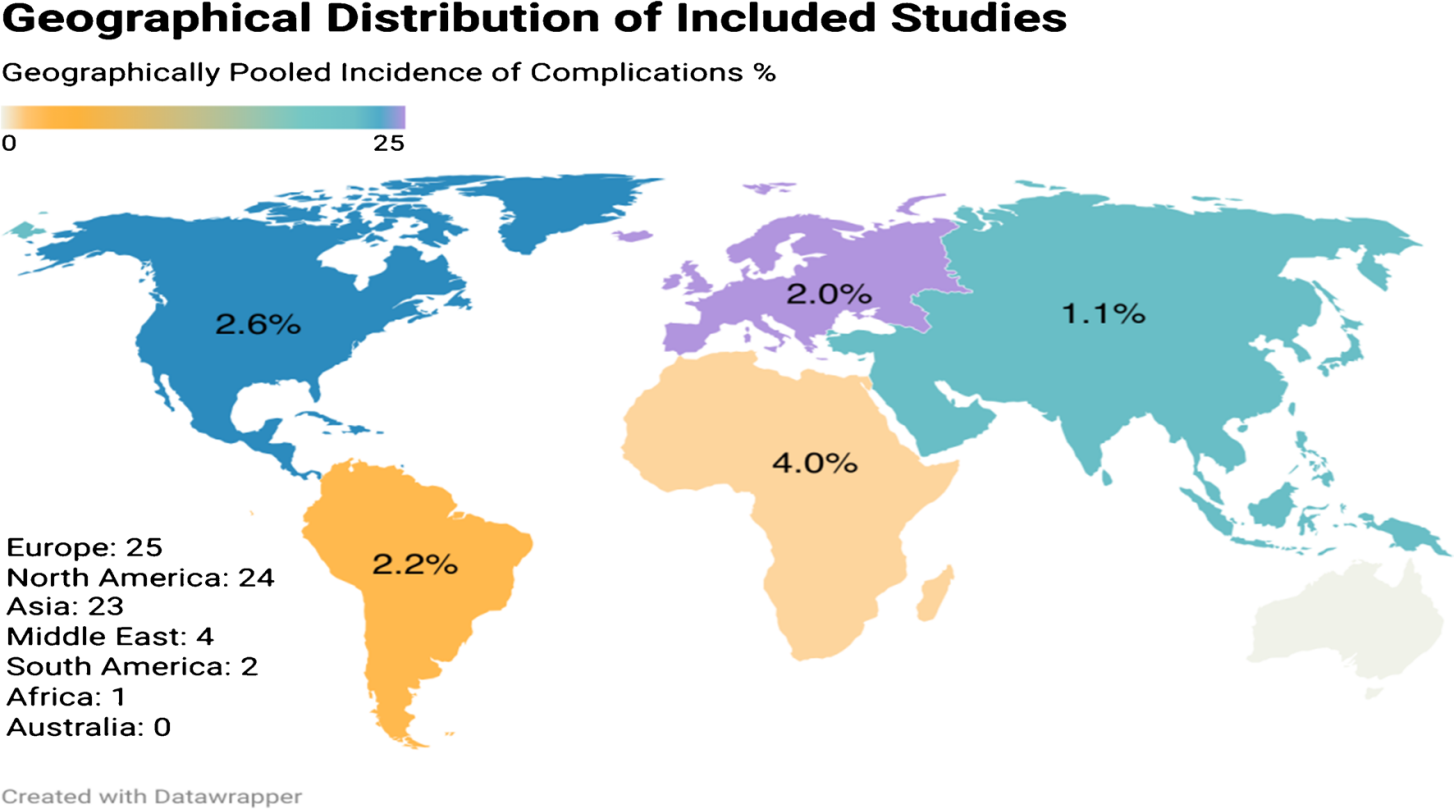
Geographic variation in publication density and complication prevalence: a random effects meta-analysis

The 79 studies included in this review originated from 26 countries. Most publications (32%) were from Europe, followed by North America (30%) and Asia with publications at 29%. Aside from Australia and Antarctica, all continents were represented. Figure 4 displays the global distribution of included studies, whilst Supplementary Fig. 9 depicts the meta-analysis.

### Correlation of anaesthetic mixture and complication rate

All studies reported using anaesthetic mixtures within the accepted dosage (7 mg/kg lidocaine), and as tumescent (subcutaneous) anaesthesia. Studies on WALANT and fracture surgery (no = 26) additionally added anaesthesia in the periosteal areas where fracture fixation was planned. Amongst 49 studies reporting the exact anaesthetic mixtures, 28 used sodium bicarbonate (NaHCO_3_; 8.4% concentration) with the anaesthetic solution, at a ratio of 1 cc NaHCO3:10 cc lidocaine solution. The addition of NaHCO_3_ was found to reduce overall complications, with a 0.53%, 95% CI: 0.12–1.1% complication rate compared to 1.3%, 95% CI: 0.12–2.1% in solutions without NaHCO_3_, a statistically significant difference (*p* = 0.025). Of the included studies, 60 reported the concentration of epinephrine, and 49 out of these 60 studies used 1% lidocaine instead of 2%. Notably, no significant difference in complication prevalence was observed between the various lidocaine concentrations (*p* = 0.23). Additionally, grouping the concertation of epinephrine into low (> 100,000), medium (1:1000–1:100,000), and high (< 1:1000) revealed no statistically significant complications between the groups (*p* = 0·23). Table 2 provides detailed anaesthetic mixture components and combined complication rates, whilst Supplemental Figs. 10, 11, and 12 display meta-analysis forest plots of sodium bicarbonate, lidocaine concentration, and epinephrine concentration, respectively.

**Table 2:** Correlation of WALANT anaesthetic mixture with complication rates

Variable	Group	*I*^2^	*K* (no. studies)	Number of complications	Sample size (*n*)	CE model (95% CI)	*p* value CE	RE model (95% CI)	*p* value RE
Lidocaine concentration		68.6%	60	144	5586		0.23		0.23
	1%	52.8%	49			0.82% [0.50–1.20%]		1.38% [0.56–2.45%]	
	2%	70.9%	11			0.26% [0.00–1.30%]		0.32% [0.00–2.17%]	
Epinephrine concentration		69.7%	62	166	5471		0.09		0.29
	Low (> 100,000)	71.9%	51			1.31% [0.92–1.75%]		1.71% [0.82–2.82%]	
	Medium (1:1000–1:100,000)	0.0%	2			0.00% [0.00–2.64%]		0.00% [0.00–2.64%]	
	High (< 1:1000)	57.1	9			0.06% [0.00–1.19%]		0.84% [0.0–4.5%]	
Sodium bicarbonate		40.2%	49	77	3,214		0.03*		0.09
	No	49.8%	21			1.29% [0.61–2.12%]		1.54% [0.35–3.26%]	
	Yes	23.8%	28			0.53% [0.12–1.11%]		0.57% [0.09–1.31%]	

*Indicates *p* < 0.05

## Discussion

This systematic review and meta-analysis, encompassing 15,595 patients from 79 publications, presents the global patient demographic profile for WALANT-based hand and upper limb surgeries. The findings support the safety of WALANT in both emergency and elective procedures. Importantly, the study identifies a significant association between WALANT mixtures containing sodium bicarbonate and a reduced risk of complications, providing evidence for the safety of WALANT in upper limb surgeries.

Prior research, such as Lipira et al.’s study of over 10,000 hand surgery cases, reported a 2.5% complication rate within the first month post-surgery, mainly under general anaesthesia [98]. Another recent review of 59,040 hand surgery procedures also found superficial wound infection as the most common complication, with a prevalence of less than 1% [99]. This is equivalent to our analysis, where superficial wound infection was the most common complication (52% of complications found, < 1% of the total number of cases). Additionally, only a single instance of irreversible necrosis was observed. This unique occurrence involved necrosis of the digit tip, which resulted from a crush injury to the distal phalanx and was treated with Kirschner wire fixation [88]. This isolated incident mitigates the initial concern of epinephrine-induced digital necrosis.

In our meta-analysis, we examined the association between WALANT solution components and complication prevalence, as a higher lidocaine concentration is at times used to enhance the anaesthetic effect but carries with it the disadvantage of higher toxicity. The addition of sodium bicarbonate has been shown to give 1% lidocaine solution an anaesthetic effect that is equivalent to that of 2% lidocaine solution whilst reducing the risk of anaesthetic toxicity [100, 101]. The primary use of sodium bicarbonate is to reduce the pain of injections by neutralizing the pH value of the injected solution, and double-blinded trials have proven that this is the case [102]. We found no difference in complication rates between 1 and 2% lidocaine solutions, but a significant difference if the lidocaine was buffered with sodium bicarbonate. In acute wound healing, as after a surgical procedure, a neutral pH has been found to optimize protease activity and cell migration, thus enhancing the potential of wound healing [103]. Given that wound healing complications were the most common in our systematic review of WALANT surgery, we recommend adding sodium bicarbonate. This addition helps lower injection pain, enhances lidocaine’s anaesthetic effect, and reduces the risk of both lidocaine toxicity and postoperative complications.

In our analysis of three common hand procedures—CTR, TFR, and FTR—we observed a complication rate of 0.05% for CTR. Notably, the largest study to date, a national cohort analysis of over 850,000 CTR cases, reported a similar overall complication rate of around 0.1% [104]. For TFR, reported complication rates vary widely, ranging from 1 to 43% [105–109]. Everding et al. found a complication rate of 12% in their review of 795 TFR, and another retrospective review of 3428 patients reported a rate of 16% of complications [110, 111]. Kardestuncer et al., in their review of 527 fingers that underwent trigger release under local anaesthesia without using epinephrine, found a complication rate of 6.3% [112]. In our systematic review, we report a 0.5% rate which is low in comparison to all previous studies and thus adds to the strength and safety of using WALANT.

Amongst traumatic hand surgery procedures, FTR is one of the most common, and whilst our subanalysis indicated a significantly higher complication prevalence for traumatic procedures (FTR) under WALANT compared to elective procedures (CTR, TFR) (*p* = 0.036), our review revealed a notably low complication rate of 1.2%. In comparison, a comprehensive review of 39 studies (3800 cases) showed a 4–6% complication rate, including reoperations, adhesions, and ruptures [113]. This favourable outcome may be attributed to the unique ability to intraoperatively test suture strength and motion, thus underscoring the continued viability of WALANT for traumatic hand surgeries. Therefore, despite the higher complication prevalence in traumatic procedures with WALANT compared to elective ones, WALANT should still be considered for traumatic hand surgeries, as it results in a lower prevalence of complications than what is typically reported in the literature without WALANT, likely due to the active intraoperative testing of suture strength and motion.

WALANT, in addition to its safety, offers a distinct advantage by facilitating direct surgeon–patient interaction and intra-operative assessment of functional. With the patient fully awake and cooperative, intraoperative motion control can be assessed without a tourniquet, which is particularly beneficial in tendon repair and transfers. This dynamic engagement allows for immediate feedback, crucial adjustments, and contributes to superior surgical outcomes, precision, functional recovery, and heightened patient satisfaction [5, 114–117].

Most of the included studies originated in the USA, akin to most of the published literature in orthopaedics [118–120].This can be attributed to the high research activity in the USA, in addition to the overall high health expenditure [118]. However, a closer look at the studies’ geographical distribution demonstrated that Europe was the primary source with 25 publications, followed by North America with 24, and Asia with 23 publications. This trend might reflect the cost-effectiveness and relative accessibility of WALANT. Seven countries (USA, Malaysia, France, Korea, UK, Canada, and Taiwan) contributed to 65% of the total publications. Most of the studies (71%) were published between 2020 and 2023. This surge can be explained by a general dramatic increase in publications during the COVID-19 pandemic [121]. In contrast, only 25% of the publications were published between 2010 and 2019, and only 4% before 2009, reflecting the increase in WALANT utilization over the last 20 years. This temporal pattern also underscores the steady increase in WALANT across the globe.

WALANT offers a cost-effective approach to delivering surgical care by eliminating the need for an anesthesiologist and the expenses related to anaesthetic drugs, monitoring equipment, pre-operative evaluations, and post-anaesthesia care. This accessibility expands the reach of surgical interventions to a wider population whilst sparing operating room and anaesthesia-related costs [22–24, 81]. By requiring fewer personnel and being suitable for office-based procedures, WALANT facilitates the timely delivery of surgical care in remote or resource-limited regions. This has the potential to bridge healthcare gaps by reaching underserved areas globally.

In the context of sustainability, the increasing problem of surgical waste presents environmental challenges. Addressing this concern is possible through alternative surgical techniques. One effective approach involves minimizing the use of general anaesthesia and its associated disposables, leading to a substantial reduction in surgical waste generation [122]. According to a recent global survey involving 876 hand surgeons, over 50% identified WALANT as a key factor in making their surgical practices more sustainable [123]. Incorporating WALANT into office-based settings enhances sustainable practices in surgery.

This study concludes that WALANT is a remarkably safe technique with a low complication rate. The success of WALANT in upper limb surgery has led to its application across diverse surgical procedures highlighting its versatility and potential to revolutionize surgical care [124–126]. Our analysis of WALANT provides evidence for its adoption in our in upper limb and hand practice, inspiring consideration by other surgical specialties and proposing WALANT as a potentially effective model for providing care in resource-constrained areas. Based on these findings, healthcare administrators and professionals are urged to recognize the transformative potential of the WALANT technique in upper limb procedures. Its noteworthy combination of low complication rates, cost-effectiveness, and high adaptability can catalyse a transformative shift in orthopaedic and plastic surgery.

We noticed outlier studies with significantly higher complication rates, likely stemming from complex procedures including digit replantation [94], stage four Dupuytren contracture [41, 43], extensor central slip tenotomy for chronic mallet finger [30], and flexor tendon repair [91] during our analysis.

## Limitation

This research study is not without its inherent limitations, which should be considered when interpreting the findings. It is important to acknowledge that the studies included in this analysis did not uniformly investigate the same complications but rather all complications reported. This heterogeneity in the outcomes assessed across the selected studies can introduce variability in the data and impact the generalizability of our findings. Moreover, the included studies exhibited discrepancies in follow-up periods, study designs, and WALANT solution concentration or mixtures, and were conducted by different surgeons within diverse patient populations, each presenting unique medical conditions. Based on the meta-analysis performed the levels of heterogeneity were always substantial (*I*^2^ > 50%) in most of the subgroup analysis, and random effects models were reported.

Moreover, subgroup analyses were performed whenever possible to discern potential patterns or trends within the data. Therefore, the findings of this study should be interpreted with a recognition of the above limitations. The number of included studies with large sample sizes and representation from various regions in the world adds strength to this systematic r.

### Supplementary Information

Below is the link to the electronic supplementary material.

Supplementary file1 (DOCX 338 KB)

## Data Availability

Data supporting this study are included within the article and/or supporting materials.
