# Supplementary material for: Complications and side effects of Wide-Awake Local Anaesthesia No Tourniquet (WALANT) in upper limb surgery: a systematic review and meta-analysis
Source: Int Orthop. 2024 Feb 17;48(5):1257–69. doi: 10.1007/s00264-024-06104-9 (PMC11001684; doi:10.1007/s00264-024-06104-9)
Supplement: Supplementary file 1 — Supplementary file1 (DOCX 338 KB) [file 264_2024_6104_MOESM1_ESM.docx]

**Supplementary Figure 1:** Electronic Database Key Terms Searched

| Database | Search queries for articles from inception to February 25, 2023, executed on February 26, 2023 |
| --- | --- |
| Web Of Science | 'wide-awake' OR 'wide awake' OR 'wide awake no tourniquet' OR 'wide awake without tourniquet' OR 'walant' OR 'local anesthetic no tourniquet' OR 'local anesthetic without tourniquet' |
| Embase | 'wide-awake' OR 'wide awake' OR 'wide awake no tourniquet' OR 'wide awake without tourniquet' OR 'walant' OR 'local anesthetic no tourniquet' OR 'local anesthetic without tourniquet' |
| Scopus | TITLE-ABS-KEY ('wide-awake' OR 'wide AND awake' OR 'wide AND awake AND no AND tourniquet' OR 'wide AND awake AND without AND tourniquet' OR 'walant' OR 'local AND anesthetic AND no AND tourniquet' OR 'local AND anesthetic AND without AND tourniquet') |
| Cochrane | 'wide-awake' OR 'wide awake' OR 'wide awake no tourniquet' OR 'wide awake without tourniquet' OR 'walant' OR 'local anesthetic no tourniquet' OR 'local anesthetic without tourniquet' |
| MEDLINE | 1. wide awake.mp. 2. wide awake no tourniquet.mp. 3. wide awake without tourniquet.mp. 4. walant.mp. 5. local anesthetic no tourniquet.mp. 6. local anesthetic without tourniquet.mp. 7. 1 or 2 or 3 or 4 or 5 or 6 or 7 or 8 |

**Supplementary Figure 2**: Forest Plot of Prevalence of WALANT Complications in Carpal Tunnel Release

**Supplementary Figure 3:** Forest Plot of Prevalence of WALANT Complications in Trigger Finger Release

**Supplementary Figure 4:** Forest Plot of Prevalence of WALANT Complications in Flexor Tendon Repair

**Supplementary Figure 5**: Forest Plot Comparing Complication Rates in Elective vs. Traumatic Procedures Under WALANT (CTR and TFR vs. FTR)

**Figure 6:** Forest Plot of WALANT Complications Over Time

**Supplementary Figure 7:** Forest Plot of Study Design and Reported Complications in WALANT

**Supplementary Figure 8:** Forest Plot of WALANT Complication Rate Stratified by Age

**Supplementary Figure 9**: Forest Plot of WALANT Geographical Distribution of Publications and Complication Rates

**Supplementary Figure 10:** Forest Plot of Sodium Bicarbonate Use in WALANT Solutions

**Supplementary Figure 11:** Forest Plot of Epinephrine Concentration in WALANT Solutions

**Supplementary Figure 12:** Forest Plot of Lidocaine Concentration in WALANT Solutions
